# *Schistosoma mansoni* among pre-school children in Musozi village, Ukerewe Island, North-Western-Tanzania: prevalence and associated risk factors

**DOI:** 10.1186/s13071-015-0997-9

**Published:** 2015-07-16

**Authors:** Deodatus M. Ruganuza, Humphrey D. Mazigo, Rebecca Waihenya, Domenica Morona, Gerald M. Mkoji

**Affiliations:** Department of Medical Parasitology and Entomology, School of Medicine, Catholic University of Health and Allied Sciences, P.O. Box 1464, Mwanza, Tanzania; Department of Zoology, Jomo Kenyatta University of Agriculture and Technology, P.O. Box 62000–00200, Nairobi, Kenya; Institute of Tropical Medicine and Infectious Diseases (ITROMID), Jomo Kenyatta University of Agriculture and Technology, P.O. Box 62000–00200, Nairobi, Kenya; Centre for Biotechnology Research and Development, Kenya Medical Research Institute (KEMRI), PO Box 54840–00200, Nairobi, Kenya

**Keywords:** *S. mansoni* prevalence, Intensity of infection, Pre-school children, Tanzania

## Abstract

**Background:**

Recent evidence indicates that pre-school children (PSC) living in *S. mansoni* highly endemic areas are at similar risk of schistosomiasis infection and morbidity as their school aged siblings. Recognizing this fact, the World Health Organization (WHO) is considering including this age group in highly endemic areas in control programmes using mass drug administration (MDA). However, detailed epidemiological information on *S. mansoni* infection among PSC is lacking for many endemic areas, specifically in Tanzania. This study was conducted to determine the prevalence of *S. mansoni* infection and its associated risk factors among PSC in Ukerewe Island, North-Western Tanzania.

**Methods:**

This was a cross-sectional study, which studied 400 PSC aged 1–6 years. The Kato-Katz (K-K) technique and the point of care circulating cathodic antigen (CCA) immunodiagnostic test were used to diagnose *S. mansoni* infection in stool and urine samples respectively. A pre-tested questionnaire was used to collect demographic data and water contact behaviour of the children from their parents/guardians.

**Results:**

Based on the K-K technique, 44.4 % (95 % CI: 39.4–49.4) pre-school children were infected with *S. mansoni* and the overall geometric mean eggs per gram of faeces (GM-epg) was 110.6 epg with 38.2 and 14.7 % having moderate and heavy intensity infections respectively. Based on the CCA, 80.1 %, (95 % CI: 76.0–84.0) were infected if a trace was considered positive, and 45.9 %, (95 % CI: 40.9–50.9), were infected if a trace was considered negative. Reported history of lake visits (AOR = 2.31, 95 % CI: 1.06–5.01, *P* < 0.03) and the proximity to the lake shore (<500 m) (AOR = 2.09, 95 % CI: 1.05–4.14, *P* < 0.03) were significantly associated with *S. mansoni* infection. Reported lake visit frequency (4–7 days/week) was associated with heavy intensities of *S. mansoni* infection (*P* < 0.00).

**Conclusion:**

The prevalence of *S. mansoni* infection in the study population using K-K and CCA-trace-negative was moderate. The frequency of lake visits and the proximity to the lake shore were associated with the infection of *S. mansoni* and its intensity. These findings call for the need to include the PSC in MDA programmes, public health education and provision of safe water for bathing.

## Background

Schistosomiasis remains a serious public health concern in sub-Saharan Africa and approximately one-third of the 192 million cases of schistosomiasis in the region are caused by *Schistosoma mansoni*, the causal agent of intestinal schistosomiasis [1]. An estimated 4.4 million people infected with *S. mansoni* have bloody diarrhoea and another 8.5 million have hepatomegaly associated with periportal liver fibrosis, portal hypertension and haematemesis [2]. Furthermore, complications of oesophageal varices and haematemesis supposedly result into 130,000 annual deaths [2, 3]. Among the school-going children, a chronic infection with *S. mansoni* contributes towards anaemia, stunted growth, organomegaly and poor cognitive functions; the combination of these morbidities may affect their future life [4, 5].

In Tanzania, studies on *S. mansoni* have been carried out mostly in school children where the prevalence of *S. mansoni* infection ranges from 32 to 64 % and varies from one geographical area to another [6–8], furthermore 46 % lack access to potable water, limiting the use to drinking where available, 87 % lack improved sanitation and 16 % practice open defaecation [9]. In the nearby East African countries the prevalence of *S. mansoni* in preschool children (PSC) was 44.3 and 16 % respectively on the shores of Lake Albert and Lake Victoria in Uganda [10] and 14 % in Lake Victoria in Kenya [11]. Although intestinal schistosomiasis has been known to infect PSC since the early 1950’s [12, 13], it is only recently that attention has focused on this particular group [10, 14–20]. For a long time, this age group was considered to be at low risk of severe infection [21]. However, given that the Kato-Katz technique, used for many years in epidemiological surveys of *S. mansoni* infection, has a low sensitivity for infection detection, the prevalence of infection among PSC may have been grossly under-estimated [22–25]. This could have resulted in the exclusion of the age group in the control programmes. It has been suggested that supplementing the Kato-Katz technique with diagnostic techniques that have a greater sensitivity such as the Circulating Cathodic Antigen test (CCA) (Sensitivity between 76.7 and 99.1 %, Specificity of 74.2 %), could improve the detection of infections in PSC [22–31]. As a matter of fact, a higher prevalence was recorded among school children in other endemic areas in sub-Saharan Africa when the CCA immunodiagnostic test was used alongside the Kato-Katz test [10, 11, 32]. However, ambiguity remains for the interpretation of CCA results where the traces in the test band can be considered either negative or positive, affecting the sensitivity of the test [33]. One study has shown a good sensitivity and specificity when traces are considered negative, but specificity is poorer when traces are considered positive [34]. It has been shown that CCA is not affected by *S.haematobium* [35] or Soil-transmitted helminths [36], while on the other hand Lewis-X trisaccharide epitopes can be picked up contributing to false positives [34]. The CCA test was used in the present study in addition to the Kato-Katz test.

Even where schistosomiasis has been investigated among PSC, very little is known about the risk factors associated with the disease in this age group. However, it has been observed that PSC can be exposed to infection when they accompany their parents/guardians or their older siblings to transmission sites [20]. Among the older children, risk factors often associated with schistosomiasis include the distance of households from the water contact points, the involvement in fishing or farming activities, gender and socio-economic status [15, 17, 18, 20, 37].

Although the World Health Organization (WHO) has acknowledged the need to include pre-school aged children in schistosomiasis control programmes, the epidemiological information on schistosomiasis among PSC remains scanty for many endemic areas, including Tanzania, where 51 % of the population is at risk of being infected [38]. The aim of the present study was to determine the prevalence and intensity of *S. mansoni* infection among PSC, aged 1 to 6 years (12–83 months) in an endemic area, the Musozi village on the Ukerewe Island, in North-Western Tanzania and to identify the risk factors associated with the infection.

## Methods

### Study area and population

**Fig. 1:**
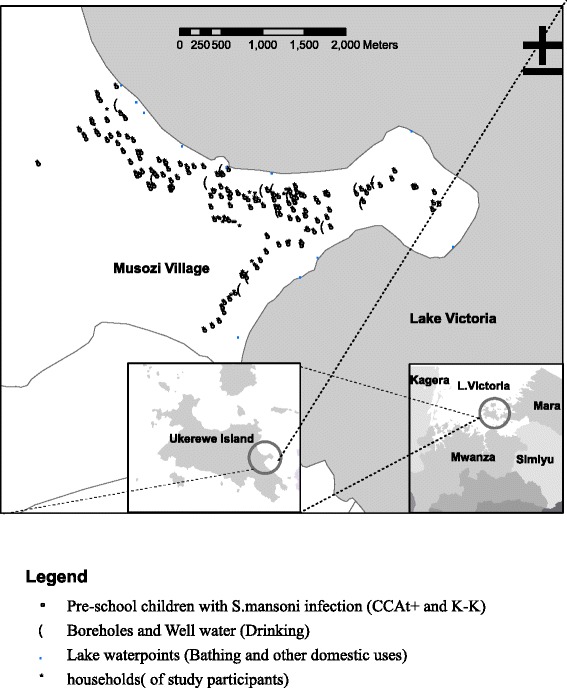
Map of Musozi village showing households of pre-school children, wells and lake water contact points

Musozi village is located on the main Island of Nansio, in Ukerewe district, Mwanza Region, at approximately 2°S and 33°E in the North-Eastern part of the island, and at an altitude of 1100 m above sea level (Fig. 1). The climate is tropical and temperatures typically range from 17 to 27 °C with a mean annual rainfall of 1090 mm. The area experiences two rainy seasons (a short rainy season between October and January, followed by a short dry spell from mid-January to February), and a long rainy season from March and June. According to the most recent census, the village has 712 PSC aged from 1 to 6 years (12 to 83 months) [39].

### Study design, inclusion and exclusion criteria

This was a cross-sectional study of 400 systematically selected PSC aged 1–6 years (12–83 months), who had lived in the study village for at least 6 months before the study began and who had no history of having been treated with Praziquantel (PZQ) in the previous 1 year. A written consent was obtained from parents/guardians of the children, prior to their enrolment into the study.

### Data collection

#### Questionnaire

A structured questionnaire was administered to the PSC’s parents or guardians, the questionnaires collected information on the demographic characteristics of the PSC and their parents’ demographic characteristics and water contact behaviour as a proxy for their children’s risk of water contact. Water contact behaviour was assessed by inquiring on history of water contact i.e. whether the child has ever been to the lake and has had previous contact with lake water, Mode of contact i.e. whether the child goes alone, with sibling or with parents/guardian, frequency i.e. the reported number of days in the last week that the child has been to the lake.

#### Mapping of the household, lake water points and wells/boreholes

Global positioning system (GPS) data was taken for the study participant household, the closest Lake water contact point (where the household members bathe and fetch water for other domestic uses) and Well water point were taken by using a Garmin etrex handheld GPS receiver (Trimble Navigation, Sunnyvale, CA, USA). Data was recorded on data sheets and was later entered into Microsoft excel 2010 and then ArcGIS ArcMap 10.2.2 were analysis was done to obtain the distance from each household and its nearest lake water contact point. Distance data were taken to Stata for further analysis and the map of the study site was generated.

#### Parasitological examination of stool samples

Single stool and urine samples were collected in separate clean containers from each of the enrolled children. From each stool sample two Kato-Katz thick smears were prepared using a template that can hold 41.7 mg of stool sample, supplied by Vestergaard Frandsen (Lausanne, Switzerland), according to a standard protocol [23, 40, 41]. The slides were left for 24 h for them to clear for easy visualization of *S. mansoni* eggs. After 24 h, the smears were examined for eggs of *S. mansoni* and other helminths. Two independent and experienced parasitologists examined each slide. For each slide, characteristic eggs of *S. mansoni* that were present were noted by examiners.

#### Qualitative examination of CCA for *S. mansoni* in urine samples

In addition to examining Kato-Katz stool smears by microscopy, urine samples were also collected from the enrolled study children and were tested for *S. mansoni* Circulating Cathodic Antigens (CCA) using the CCA Urine Cassette assay (Rapid Medical Diagnostics, Pretoria, South Africa) [42]. The preparation and examination of the urine samples were performed according to the manufacturer’s instructions [42]. The results of the test were read 20 min after adding the buffer. If the control bands did not develop, the result was regarded as invalid. Because there is ambiguity in the interpretation of ‘trace’ in a CCA test band i.e. it can be read as positive or negative [33], we used two categories for diagnostic analysis in which valid results were either scored as trace positive or trace negative.

### Ethical considerations

This study was approved by the Kenya Medical Research Institute (KEMRI) Scientific and Ethics Committee (SSC No. 2739) and by the Joint Institutional Review Board of the Catholic University of Health and Allied Sciences (CUHAS) and Bugando Medical Centre, Mwanza, Tanzania (review and publication certificate number: BREC No. 035/2013). Prior to recruiting the study participants, village meetings were held with parents and guardians, under the guidance of village leaders to explain the objectives of the study and the procedure to be used for data collection. Written informed consent was obtained from the parents/guardians of children before their enrolment into the study. Stool and urine collection are routine procedures and do not pose any risks to the study children. Praziquantel and albendazole at the recommended doses were administered to the infected children under the supervision of a qualified clinician. All the information obtained from the children and their parents was treated as private and confidential and the records were stored in a locked cabinet and data were entered in a password protected file in a computer.

### Data analysis

The data entered and stored in Microsoft Excel 2007 was checked for entry errors and was analysed using statistical tools available in the STATA version 12 software (STATA Corp, College Station, Texas, USA). The age variable was described as mean ± standard deviation and categorized into age groups: 1- < 2, 2- < 3, 3- < 4, 4- < 5, 5- < 6, 6- < 7 years. Binary variables were compared using the Chi-square test (*χ*^2^) or the Fisher exact test, where appropriate. The intensity of infection was categorized according to WHO criteria as ≤99 epg (light), 100–399 epg (moderate), or ≥400 epg (heavy) [43, 44]. Prevalence of infection was of <10 % is low, ≥10 but <50 % is moderate prevalence and >50 % high prevalence. ANOVA or t-tests were used to test for the difference in mean egg counts for *S. mansoni* between sex and age-groups.

Demographic and socio-economic variables were assessed using logistic regression. The potential associations were first assessed at a bivariate level; then, factors with a *P*-value <0.2 were entered into the multivariate model. Sex and age group term were entered as *a priori* into the multivariate model. Stepwise backward logistic regression was used to determine whether these variables were independent factors of *S. mansoni*. Independent risk factors of faecal egg counts were identified in a linear regression model using a log-transformed egg per gram of faeces (epg) as outcomes variable and social demographic factors as explanatory variables. The odd ratios of each of the risk factors associated with faecal egg counts were obtained by taking the antilogarithm of the regression coefficient.

## Results

### Demographic characteristics of the study children and prevalence of *S. mansoni* infection

A total of 400 PSC aged 1–6 years (12–83 months) were enrolled into the study. Of these children, 49.2 % (*N* = 197) were female and 50.7 % (*N* = 203) were males. The mean age of the children was 3.82 ± 1.6 years. Table 1 shows the age and sex distribution of the children.

**Table 1:** Demographic characteristics of 400 pre-school children from Musozi village, in Ukerewe district, north-Western Tanzania

Age (years)	Males	%	Females	%
1- < 2	26	12.81	37	18.78
2- < 3	36	17.73	41	21.81
3- < 4	42	20.69	28	14.21
4- < 5	30	14.78	31	15.74
5- < 6	39	19.21	35	17.77
6- < 7	30	14.78	25	12.69
Total	203		197	

### Prevalence of *S. mansoni* based on the Kato-Katz technique

**Fig. 2:**
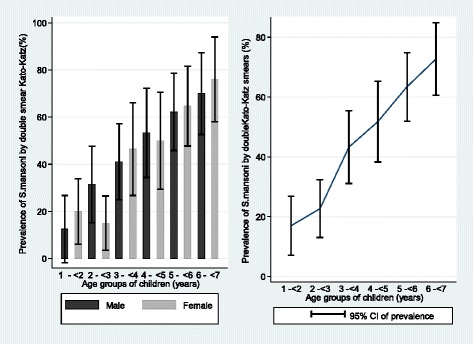
Prevalence of *S. mansoni* based on Kato Katz technique among 383 pre-school children of Musozi villages, north-western Tanzania, stratified by sex and age

Based on the Kato-Katz stool smear results, a total of 170/383 children, 44.4 %, (95 % CI: 39.4–49.4) were found to be infected with *S. mansoni*, with a prevalence in males being 46.2 % (90/195), (95 % CI: 39.0–53.2), and in females 42.6 % (80/188), (95 % CI: 39.4–49.7). No gender-related difference in prevalence was observed (*χ*^2^ = 0.50, *P* < 0.48). The prevalence of infection increased with age (*χ*^2^ = 61.8, *P* < 0.00), with the youngest age group, 1- < 2 years, exhibiting a moderate prevalence of 16.9 % and the much older age group, 6- < 7 years, a high prevalence of 72.7 %. The prevalence of infection was highest (>50 %) in the age group 4- < 5 years. The prevalence of *S. mansoni* infection based on the Kato-Katz stool smear results stratified by age and sex is shown in Fig. 2.

### Prevalence of *S. mansoni* based on Circulating Cathodic Antigen (CCA)

**Fig. 3:**
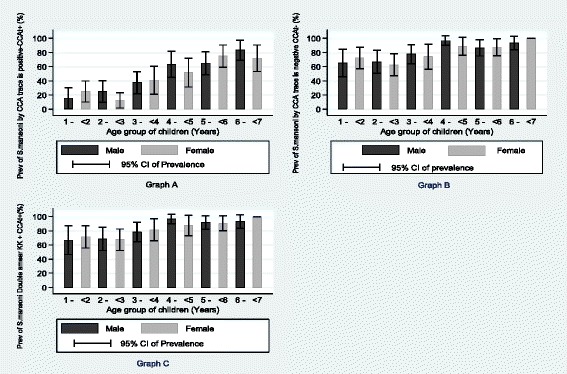
Prevalence of *S. mansoni* based on circulating cathodic antigen, **a**: not including trace and **b**:including trace and **c**: combined Kato Katz and CCA trace positive among 383 pre-school children of Musozi villages, north-western Tanzania, stratified by age

The prevalence of *S. mansoni* infection based on Point of Care-Circulating Cathodic Antigen Urine (POC-CCA) cassette tests, stratified by age and sex, is shown in Fig. 3. With regards to the POC-CCA, considering a trace as a positive result (CCAt+), 309/386, 80.1 % (95 % CI: 76.0–84.0) of the PSC were infected with *S. mansoni*. Stratified by age, the prevalence of *S. mansoni* in male children was 80.9 % (95 % CI: 75.3–85.3), while the prevalence for females was 79.1 % (95 % CI: 73.3–85.0). Stratified by age, the prevalence of *S. mansoni* (CCAt+) was 69.3, 64.5, 76.1, 93.0, 87.0 and 96.0 % for age groups 1- < 2, 2- < 3, 3- < 4, 4- < 5, 5- < 6, 6- < 7 years respectively. Considering a trace as a negative result (CCAt-), 177 out of 386 were found positive for *S. mansoni* infection and the prevalence was 45.9 % (95 % CI: 40.9–50.9). The prevalence of *S. mansoni* in male children was 48.2 % (95 % CI: 41.2–51.2) while the prevalence in female children was 43.3 % (95 % CI: 36.2–50.5). Stratified by age, the prevalence of *S. mansoni* was 21.0, 18.4, 38.8, 57.9, 69.6 and 78.2 % for age groups 1- < 2, 2- < 3, 3- < 4, 4- < 5, 5- < 6, 6- < 7 years respectively.

### Overall prevalence of *S. mansoni* based on Kato-Katz technique and circulating cathodic antigen combined

The overall prevalence of *S. mansoni* infection based on the combined results of Kato-Katz technique and CCAt + was 82.1 % (95 % CI: 78.2–86.0). The prevalence of *S. mansoni* infection did not vary by sex (*χ*^2^ = 0.12, *P* < 0.72), with male and females having a prevalence of 82.8 and 81.4 % respectively. However, in relation to age, there was a significant difference in the prevalence of *S. mansoni* infection (*χ*^2^ = 32.3, *P* < 0.00) with the youngest age group having a prevalence of 69.5 % and the older age group having a prevalence of 96.4 %.

### Intensity of *S. mansoni* infection

**Fig. 4:**
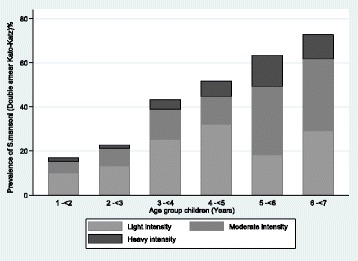
Prevalence and intensities of *S. mansoni* based on Kato Katz technique among 383 pre-school children of Musozi villages, north-western Tanzania, stratified by age

The distribution of intensity of *S. mansoni* infection by age and sex is shown in Table 2 and The intensity category of *S. mansoni* infection is shown in Fig. 4. The overall geometric mean egg per gram of faeces (GM-epg) for the study participants was 110.61 GM-epg (95 % CI: 93.1–131.4). Male children had a higher GM-epg, 119epg (95 % CI: 93.7–150.8) than female children 102 epg (95 % CI: 79.2–131.4). However, the difference observed did not reach significance (t = 0.83, *P* = 0.79). The GM-epg was observed to increase with age, the older age group had the highest GM-epg (F = 6.38, *P* < 0.00). Of the 170 children with detectable *S. mansoni* eggs, 47.1, 38.2 and 14.7 % had light, moderate and heavy infections respectively, as defined by WHO [44].

**Table 2:** The distribution between the geometric mean intensity of infection in preschool children with *S.mansoni* infection in Musozi village by age and sex

Age category (years)	Male GMI (epg)	95 % CI	Female GMI (epg)	95 % CI	Combined GMI (epg)	95 % CI
1- < 2	283.9	25.5–2122.8	45.8	15.8–134.8	74.7	29.7–189.6
2- < 3	94.9	39.3–228.9	46.0	10.8–195.5	73.5	39.9–146.4
3- < 4	96.9	58.5–160.4	62.4	33.6–115.5	79.5	54.6–115.8
4- < 5	85.2	46.2–157.2	100.8	51.5–197.2	90.9	60.1–140.4
5- < 6	148.7	94.5–234	209.5	133.9–327.6	175.8	128.9–239.7
6- < 7	143.3	80.5–255	108.0	71.7–162.7	125.3	88.6–117.2

### Factors associated with *S. mansoni* infection in pre-school children

The association between the prevalence of *S. mansoni* infection and demographic characteristics, water contact patterns and distance from the lake water contact point are shown in Table 3. Age (*P* < 0.01), history of going to the lake (*P* < 0.01), mode of contact (*P* < 0.01), frequency of lake visits (*P* < 0.01) were significantly associated with *S. mansoni* infection.

**Table 3:** Prevalence of infection with *S.mansoni* in pre-school children in Musozi village in relation to demographic characteristics water contact patterns and distance from the lake water contact point

Characteristic	No examined	No infected (Prevalence)	95 % confidence interval	*χ*2 *P* value
Sex				
Female	183	149 (81.42)	75.73–87.11	<0.73
Male	192	159 (82.81)	77.43–88.20	
Age (years)				
1- < 2	59	41 (69.45)	57.39–81.59	<0.00
2- < 3	75	51 (68.00)	57.19–78.80	
3- < 4	64	51 (79.69)	69.56–89.82	
4- < 5	54	50 (92.59)	89.38–99.80	
5- < 6	68	62 (91.18)	84.26–98.09	
6- < 7	55	53 (96.36)	91.25–101.47	
Go to the lake				
No	94	58 (61.70)	7.10–14.41	<0.00
Yes	279	249 (89.25)	85.58–92.90	
Mode of contact				
Never	97	59 (60.82)	50.94–70.71	<0.00
Accompanied	255	227 (89.02)	85.16–92.88	
Alone	21	21 (100)		
Frequency				
Never	206	148 (71.84)	65.65–78.04	<0.00
Moderate	59	51 (86.44)	77.44–95.44	
High	105	105 (100)		
Who				
Father	7	6 (85.71)	50.76–120.67	<0.68
Mother	89	85 (95.51)	91.11–99.89	
Self	10	10 (100)		
Guardian	6	6 (100)		
Sibling	49	46 (95.03)	86.92–100.83	
Distance				
≤ 500 m	146	117 (80.14)	73.58–86.68	<0.26
> 500 m	154	131 (85.06)	79.37–90.75	

Independent factors associated with the prevalence of infection are shown in Table 4. In a multivariate model, history of going to the lake (AOR = 2.13, 95 % CI: 1.06–5.01, *P* < 0.03) and living at a distance of less than 500 m from the lake water contact point (AOR = 2.09, 95 % CI: 1.05–4.14, *P* < 0.03) were associated with the infection with *S. mansoni*.

**Table 4:** The factors associated with the prevalence of infection in preschool children in Musozi village Ukerewe district

Characteristic	Bivariate			Multivariate		
	OR	95 % CI	*P*-value	AOR	95 % CI	*P*-value
Sex						
Female	1			1		
Male	1.09	0.65–1.87	0.73	1.02	0.51–2.03	0.95
Age (years)						
1- < 2	1			1		
2- < 3	0.93	0.45–1.94	0.85	0.64	0.26–1.61	0.35
3- < 4	1.72	0.76–3.92	0.20	1.11	0.40–3.10	0.83
4- < 5	5.49	1.72–17.49	0.00	2.56	0.61–10.71	0.20
5- < 6	4.54	1.66–12.78	0.00	1.32	0.37–4.62	0.66
6- < 7	11.63	2.55–53.01	0.00	2.28	0.41–12.46	0.34
Go to the lake						
No	1			1		
Yes	5.15	2.94–9.04	0.00	2.31	1.06–5.01	0.03
Mode of contact						
Never	1					
Accompanied	5.22	2.96–9.20	0.00			
Alone						
Frequency						
Never	1			1		
Moderate	2.50	1.11–5.58	0.03	1.54	0.54–4.37	0.409
High						
Who						
Father	1					
Mother	3.54	0.34–36.8	0.29			
Self						
Guardian						
Sibling	2.56	0.22–28.67	0.45			
Distance						
> 500 m	1			1		
≤ 500 m	1.59	0.84–2.98	0.14	2.09	1.05–4.14	0.034

*OR* odds ratio, *AOR* adjusted odds ratio, *CI* confidence interval

The association between the intensity of *S. mansoni* infection, demographic characteristics, water contact patterns and distance from the lake water contact is shown in Table 5. Age (*P* < 0.00), history of going to the lake (*P* < 0.00), mode of contact (*P* < 0.00) and frequency of lake visits (*P* < 0.00) were significantly associated with *S. mansoni* infection.

**Table 5:** The relationship between the geometric mean of intensity of infection (GMI) in preschool children in Musozi village in Ukerewe North Western and the demographic characteristics

Characteristic	No infected	GMI	95 % CI	*P*-values
Sex				
Male	80	101.9	79.2–131.4	<0.41^a^
Female	90	118.9	93.7–150.8	
Age				
1- < 2	10	74.7	29.4–189.6	<0.00^b^
2- < 3	17	73.5	36.9–146.4	
3- < 4	29′	79.5	54.6–115.8	
4- < 5	29	91.9	60.1–140.4	
5- < 6	45	175.8	128.9–235.7	
6- < 7	40	125.3	88.6–177.2	
Go to the lake				
No	21	77.6	45.2–133.3	<0.00^a^
Yes	149	116.3	97–139.4	
Mode of contact				
Never	21	77.6	45.2–133.3	<0.00^b^
Accompanied	136	109.7	90.6–132.8	
Alone	13	213.6	124.4–366.6	
Frequency				
Never	57	75.7	55.2–104	<0.00^b^
Moderate (1–3)	33	93.5	66.9–130.5	
High (4–7)	76	117.9	117.9–193.8	
Who				
Father	5	101	46.3–220.5	
Mother	34	133.7	92–194.3	
Self	62	122.7	93.3–161.5	
Guardian	6	350.7	137.9–892	
Sibling	3	122	7.6–1967	
Distance				
> 500 m	70	123.1	92.8–163.1	<0.28^a^
≤ 500 m	71	108.9	83–142.9	

^a^Determined by *T*-test

^b^Determined by ANOVA

The independent factors associated with log-transformed egg counts of *S. mansoni* are shown in Table 6*.* In a multivariate model, high frequency (4–7 days per week) of going to the lake (OR = 1.84,1.18–2.86, *P* < 0.00) remained associated with a heavy intensity of infection.

**Table 6:** The factors associated with log transformed egg counts in preschool children in Musozi village Ukerewe district North Western Tanzania

Characteristic	Bivariate			Multivariate		
	OR	95 % CI	*P*-value	AOR	95 % CI	*P*-value
Sex						
Female	1			1		
Male	1.16	0.83–1.64	0.38	1.17	0.83–1.65	0.13
Age (years)						
1- < 2	1					
2- < 3	0.98	0.41–2.34	0.97	0.94	0.39–2.24	0.88
3- < 4	1.06	0.47–2.36	0.87	0.98	0.43–2.26	0.97
4- < 5	1.23	0.55–2.73	0.60	1.10	0.47–2.56	0.81
5- < 6	2.35	1.10–5.03	0.02	2.05	0.90–4.69	0.08
6- < 7	1.67	0.78–3.62	0.19	1.49	0.65–3.41	0.33
Go to the lake						
No	1			1		
Yes	1.49	0.89–2.51	0.12	0.74	0.39–1.41	0.36
Mode of contact						
Never	1					
Accompanied	1.41	0.84–2.37	0.19			
Alone	2.75	1.26–6.00	0.01			
Frequency						
Never	1			1		
Moderate	1.23	0.76–1.98	0.38	1.30	0.77–2.19	0.30
High	1.99	1.36–2.91	0.00	1.84	1.18–2.86	0.00
Who						
Father	1					
Mother	1.20	0.26–5.57	0.80			
Self	1.21	0.46–3.21	0.69			
Guardian	1.32	0.48–3.60	0.58			
Sibling	3.47	0.97–12.34	0.05			
Distance						
> 500 m	1					
≤ 500 m	0.88	0.60–1.30	0.53			

### Spatial distribution of *S. mansoni* infection, wells and lake water contact points

The map of Musozi village showing the households of where PSC live showing distribution of *S.mansoni* infection, the lake water and well water contact points is shown in Fig. 1. The village has 12 boreholes/wells that are evenly distributed providing village members with easy access to well water; however, the water is used solely for drinking. The village also has 11 lake water contact points (*S. mansoni* infection sources) used to fetch water for other domestic needs including bathing.

## Discussion

In the present study area, it appears that *S. mansoni* infection starts at an early age and is associated with various risk factors. This adds to the growing body of evidence that PSC can have schistosomiasis. The prevalence was observed to vary by age groups but not by sex. Infections with *S. mansoni* were predominantly light, but moderate intensity and heavy intensity infections were increasingly becoming common from the age of 3 years. The main identified risk factors associated with *S. mansoni* infection in PSC were history of water contact and living at a distance of less than 500 m from the lake water contact point. A bathing frequency at the lake of 4–7 days per week was associated with a heavier intensity of infection.

The prevalence of egg patent *S. mansoni* infection was 44.39 % based on Kato-Katz, 45.85 % based on CCA when trace was considered as negative and 80.05 % on CCA when trace was considered as positive, of all the PSC who participated in the study. The prevalence estimates as determined by single stool double smear Kato-Katz and CCA with trace considered as negative (CCAt-) are broadly similar, however, the 35 % increment in prevalence observed when trace is considered as positive (CCAt+) is noted. It has been previously noted that when assessing the prevalence of *S. mansoni* using the CCA test alongside K-K, there is a higher ‘true’ prevalence with CCA than observed prevalence by K-K rather than the CCA test yielding a high number of false positives [33]. The ‘traces’ for this study could partly be accounted for by the fact that in this population most of the children have light infection as noted by the Kato-Katz, therefore, it is possible that the traces can be explained by pre-patent infections.

The overall prevalence of *S. mansoni* observed in this study was higher than that reported on the same age-group in Uganda on the shores of Lake Albert and Lake Victoria [45], in Usoma, Kenya [11] and in Ivory Coast [32] in West Africa. In addition, the overall geometrical mean in the present study was of moderate intensity and the majority of the children had light to moderate infection intensities. Only 6.53 % of the study participants most of them aged three years and above presented with heavy intensities. The intensity of *S. mansoni* infection was observed to increase with an increase in the age of the study participants. The results of the present study also show that there was no significant difference in intensity of infection by sex. This is likely due to a similarity of PSC’s water contact behaviour and parents’ or guardians’ behaviour towards their children’s water contact between the two sexes. As they grow older this similarity is lost as their behaviour towards water contact changes. This is affirmed by higher prevalence and intensity of *S. mansoni* in males than females in school children. Similar findings were reported in Ivory Coast [32]. In comparison to studies conducted on the shores of the Lake Victoria in Uganda [45] and West Africa [32], the prevalence of heavy infection among PSC was higher in the present study. This could be due to the behavior of this population group especially those living 500 m from the lake and their repeated contact with contaminated water and lack of safe water for bathing and improved sanitation facilities. Adult individuals living in this particular setting have been repeatedly reported to be highly infected with *S. mansoni* and carry the highest intensities of infection [46, 47]. Increased water contact activities such as fishing, bathing and farming were associated with an increased risk of being infected with *S. mansoni* in the adult population [48].

One remarkable observation was that heavy infections were more common from the age of three years onwards where the prevalence was 4.4 % in 3-year-olds and 10.9 % in 6-year-olds. This has shown that young children can have heavy infections even at an age as young as 3 years old. It has been depicted that the heavier the infection the more severe the morbidity; however, even light egg patent infections [49, 50] and sub-egg patent infections are considered to be detrimental to well-being, especially in younger children [51, 52]. It is therefore important to focus on these children because they are set to suffer significant morbid effects as explained by King and Dangerfield-Cha [4] and mostly because it can take up to 3 years before they are treated.

Our study also examined the risk factors associated with *S. mansoni* infection in PSC, whereby the results show a significant association between the history of a visit to the lake and the infection with *S. mansoni* in this age group. Those who reported going to the lake had twice the odds of being infected with *S. mansoni* infection. This is supported by an observation made in western Kenya where reported water contact with the lake was associated with the infection with *S. mansoni* [53]. The distance from the water contact point was strongly associated with the infection with schistosomiasis, whereby, those who lived <500 m from the lake water contact point were at twice the odds of being infected with *S. mansoni* infection. It has been demonstrated that for this age group, the distance from the water point and the history of visiting the lake were significant factors for schistosomiasis infection. This is particularly important as lake water, which is the source of water for bathing and other domestic uses including drinking for some of the villagers, was continuously contaminated with *S.mansoni* eggs from the poor sanitation practice of defaecating in the lake water. This corroborates studies carried out in Western Kenya [53] and Tanzania [6] which found that the prevalence of infection decreases with the increase of the distance from the lake water shore. A history of a high frequency of bathing in lake water, (4–7 days) per week, was shown to be associated with a heavy *S. mansoni* infection where those who had high bathing frequency in lake water were at a 1.8 higher odds of heavy infection. Our present study is subject to limitation. The findings of this study only apply to the geographical setting where the study was undertaken and cannot be generalised to other geographical settings. The temporal relationship between some of the risk factors and the study outcomes could not be assessed due to the study design.

## Conclusions

In conclusion, this study has demonstrated that the prevalence of *S. mansoni* in PSC in Musozi village in Ukerewe based on KK and CCAt- was moderate and high on CCAt+. It has also shown that moderate and heavy infections are common from the age of three years onwards. It is, therefore, important to consider including children of 3 years in PZQ-MDA programmes because they are set to suffer the significant morbid effects of chronic schistosomiasis. This study has also shown that going to the lake and living at a distance of ≤500 m to the lake were associated with a significant risk of being infected. It is important to provide health education for behaviour change to avoid contaminated water for the whole village community. Provision of safe water for the whole community for all domestic uses including bathing is also necessary. As efforts to develop and evaluate paediatric formulations of Praziquantel are underway, more studies to quantify the prevalence of *S. mansoni* infection in that particular age group should be carried out at the country level. Further investigation to quantify the morbidity due to intestinal schistosomiasis in this age group is recommended.
